# Capsaicin Ameliorates the Loosening of Mitochondria-Associated Endoplasmic Reticulum Membranes and Improves Cognitive Function in Rats With Chronic Cerebral Hypoperfusion

**DOI:** 10.3389/fncel.2022.822702

**Published:** 2022-03-17

**Authors:** Mengqi Ouyang, Qi Zhang, Jiahui Shu, Zhiqiang Wang, Jin Fan, Ke Yu, Lei Lei, Yuxia Li, Qingsong Wang

**Affiliations:** ^1^Department of Neurology, The General Hospital of Western Theater Command, Chengdu, China; ^2^Department of Pharmacology, Gaoping District People’s Hospital of Nanchong, Nanchong, China; ^3^Department of Pharmacology, Yichang Yiling Hospital, Yichang, China; ^4^Department of Neurology, Chengdu BOE Hospital, Chengdu, China

**Keywords:** vascular cognitive impairment, chronic cerebral hypoperfusion, mitochondria associated endoplasmic reticulum membranes, hippocampus, capsaicin

## Abstract

Based on accumulating evidence, vascular factors contribute to cognitive decline and dementia. Mitochondrial dysfunction is the core pathophysiological mechanism. Mitochondria-associated endoplasmic reticulum membranes (MAMs) are subcellular structures that physically and biologically connect mitochondria with the endoplasmic reticulum (ER) and regulate multiple functions ranging from calcium transfer to mitochondrial dynamics and bioenergetics. MAMs dysfunction has been speculated to be a key factor contributing to the pathogenesis of cognitive disorders and a new therapeutic target. However, the alteration of MAMs in vascular cognitive impairment remains to be revealed. Capsaicin, a specific agonist known to activated the transient receptor potential vanilloid type 1 (TRPV1), is involved in hippocampal synaptic plasticity and memory, but the detailed mechanism is still unclear. In this study, chronic cerebral hypoperfusion (CCH) model rats were created by bilateral common carotid artery occlusion (BCCAO), which is a widely used model to study vascular dementia. We observed that CCH rats showed obvious cognitive deficits, and ER-mitochondria contacts were loosener with lower expression of mitofusin2 (MFN2), a key protein connecting MAMs, in the hippocampal CA1 region, compared to the sham group. After capsaicin treatment for 12 weeks, we found that cognitive deficits induced by CCH were significantly alleviated and loosened ER-mitochondrial interactions were obviously improved. In conclusion, the findings of this study highlight that MAMs may contribute to the pathogenesis of cognitive impairment induced by CCH, and our new evidence that capsaicin improves cognitive function highlights a novel opportunity for drug discovery.

## Introduction

Vascular cognitive impairment (VCI) has become the most common cause of dementia after Alzheimer’s disease (AD) (Wu et al., 2016). A strong linkage between vascular mechanisms and cognitive deficits has been consistently acknowledged in preclinical and clinical studies (O’Brien and Thomas, 2015; Thomas et al., 2015; Wolters et al., 2017; Fouda et al., 2019). In addition, vascular pathology is a likely pathogenic contributor to dementia including AD (Sweeney et al., 2019; Cortes-Canteli and Iadecola, 2020). VCI refers to all forms of cognitive disorder associated with cerebrovascular factors, ranging from mild cognitive impairment to dementia (Dichgans and Leys, 2017). Stroke is one of the most common cerebrovascular diseases and a leading cause of death and disability (Strong et al., 2007; Hachinski et al., 2019). In addition to physical disabilities, approximately one-third of stroke survivors suffer from poststroke cognitive impairment (PSCI) and poststroke depression (PSD) (Robinson and Jorge, 2016; Mijajlovic et al., 2017), and thus they cannot return to normal social life.

Chronic cerebral hypoperfusion (CCH) is considered a fundamental pathophysiological change common to both AD and VCI (Duncombe et al., 2017; Yang et al., 2017). CCH created by bilateral common carotid artery occlusion (BCCAO) has been widely used to study VCI (Choi et al., 2011; Back et al., 2017; Washida et al., 2019; Fang et al., 2021). We previously confirmed that CCH could cause significant learning and memory impairment, especially 24 weeks after BCCAO (Wang et al., 2016; He et al., 2019). Accumulating evidence suggests that cognitive impairment induced by CCH is related to multiple pathophysiological processes including white matter injury, microvascular inflammation, oxidative stress, demyelination, blood-brain barrier (BBB) and blood-spinal cord barrier (BSCB) leakage, neurovascular unit (NVU) disruption (Du et al., 2017; Duncombe et al., 2017; Yang et al., 2017; Uemura et al., 2020), especially mitochondrial dysfunction (Yu et al., 2012; Du et al., 2017).

Mitochondria are metabolic signaling centers, contributing to adenosine triphosphate (ATP) production and multitude cellular responses such as autophagy and apoptosis (Nunnari and Suomalainen, 2012). A vital role of mitochondrial dysfunction has been demonstrated in brain aging and neurodegeneration (Sas et al., 2007; Grimm and Eckert, 2017). In our previous study, we observed that mitochondrial structural damage and mitochondrial DNA abnormalities in the hippocampal CA1 region of CCH (He et al., 2019). Mitochondria are dynamically connected to the endoplasmic reticulum (ER) via mitochondria-associated endoplasmic reticulum membranes (MAMs) (Hayashi et al., 2009; Csordás et al., 2018). As a physical and biological bridge between the two organelles, MAMs play crucial roles in various essential cellular events, such as Ca^2+^ transport, lipid metabolism, reactive oxygen species (ROS) generation and activity, bioenergetics and apoptosis (Rowland and Voeltz, 2012; Wu and Carvalho, 2018). These different functions regulated by MAMs occur varying degree of damage in neurodegenerative diseases associated with cognitive decline (Paillusson et al., 2016). Recent studies of different model indicated a potential connection between MAMs and cognitive function, including AD, Parkinson’s disease (PD), Huntington’s disease (HD), amyotrophic lateral sclerosis (ALS) and frontotemporal dementia (FTD) (Stoica et al., 2016; Area-Gomez and Schon, 2017; Paillusson et al., 2017; Cherubini et al., 2020). Accordingly, we hypothesized that there might be some common characteristics associated with MAMs in cognitive decline. However, no evidence of MAMs alterations has been demonstrated in VCI, and important pieces are still missing from the puzzle that explain how MAMs are involved in cognitive impairment.

In addition, it has been reported that CCH could induce a chronic and significant decrease in cerebral blood flow (CBF), which also contributes to the pathogenesis of VCI (Jing et al., 2015; Vidyanti et al., 2020). Glial cells, particularly astrocytes, part of the NVU, communicate with neurons and blood vessels to maintain the function of the BBB and BSCB, and contribute to CBF control (Kisler et al., 2017; Liu et al., 2020). Moreover, dysregulation of water homeostasis in the central nervous system (CNS) mediated by glial cells appear to play critical and interactive roles regarding the pathophysiology of neurovascular dysfunction of neurodegenerative diseases (NDs) and ischemia such as stroke, and targeting aquaporin 4 (AQP4) channels might be a potential therapeutic strategy (Kitchen et al., 2020; Sylvain et al., 2021).

Indeed, no specific treatments are available for cognitive decline (Montine et al., 2021). Most treatment strategies aim to prevent neuronal loss or protect neuronal circuits, even replace it (Qian et al., 2020). Capsaicin, a specific agonist of the transient receptor potential vanilloid type 1 (TRPV1) (Yang et al., 2015), has been reported to protect hippocampal synaptic plasticity and spatial memory retrieval and to protect against ischemia (Pegorini et al., 2005; Huang et al., 2017; Du et al., 2020; Wang et al., 2020a). TRPV1, also known as vanilloid receptor subtype 1 (VR1), is widely expressed in various brain areas (Caterina et al., 1997), including all cortical areas, several regions of the limbic system (e.g., the hippocampus), the striatum, the hypothalamus, the centromedian and paraventricular thalamic nuclei and the locus coeruleus, among others. TRPV1 activation, sensitively and selectively by capsaicin, has been reported to preserve hippocampal functions and spatial memory retrieval from the effect of acute stress, holding a potential to facilitate long-term potentiation (LTP) and suppress long-term depression (LTD) (Li et al., 2008). Evidence from other studies also suggested that capsaicin rescues cognitive deficits by inhibiting GluA2-containing α-amino-3-hydroxy-5-methyl-4-isoxazolepropionic acid receptor (AMPAR) endocytosis and abolishing the inhibition of protein phosphatase 2A in AD models (Jiang et al., 2013; Du et al., 2020). In astrocytes, opening of TRPV4 interact with AQP4 lead to activation of an adenylyl cyclase, production of cAMP, and activation of PKA (Kitchen et al., 2020). Activation of TRPV1 by capsaicin was recently shown to alleviate mitochondrial dysfunction in podocytes, accompanied by reduced formation of ER-mitochondria contacts and Ca^2+^ transport, in model of diabetic nephropathy (DN) (Wei et al., 2020). However, few studies elucidate the alteration of MAMs in brain under TPRV1 activation. And, to date, the specific mechanism underlying cognitive improvements mediated by capsaicin remains unclear.

Based on this evidence, we hypothesized morphological and functional changes in MAMs might occur in CCH model and capsaicin would exert a neuroprotective effect on cognitive deficits induced by CCH. We sought to analyze whether this protective effect was associated with MAMs. We focused on the hippocampal CA1 region, which play key roles in behavior and memory.

## Materials and Methods

### Animals

All animal care and experimental protocols were approved by the Animal Experiment Committee of The General Hospital of Western Theater Command and performed in accordance with the National Institutes of Health Guide for the Care and Use of Laboratory Animals. Adult male Sprague-Dawley rats weighing 280 ± 20 g were obtained from Chengdu Dossy Biological Technology Co., Ltd. [Certificate Number: SCXK (CHUAN) 2015-030, Chengdu, China].

All rats were randomly assigned into four groups (*n* = 45 rats per group) as follows: chronic cerebral hypoperfusion group underwent BCCAO (CCH), sham-operated group without BCCAO (sham), capsaicin-treated group underwent BCCAO and injected with capsaicin (capsaicin), and vehicle control group underwent BCCAO and injected with solvent (control). Each group was randomly divided into three subgroups (4, 12, and 24 weeks). Each animal was submitted to all tests. All rats were habituated for at least 7 days before the experiments. Animals were housed in groups of five per standard cage in the same animal facility maintained at a constant temperature (25 ± 2°C) and humidity (40–60%), with a 12-h light/dark cycle (lights on at 07:00 AM) and free access to food and water throughout the study period.

### Surgery

The surgical procedures to establish BCCAO were performed as described in our previously studies (Wang et al., 2016; He et al., 2019). Briefly, the rats were anesthetized with 0.15% pentobarbital sodium (P3761, Sigma, 40 mg/kg) by intraperitoneal injection. After creating a midline incision in the neck, common carotid arteries were exposed and carefully separated from the carotid sheath, cervical sympathetic and adjacent vagal nerves. Bilateral common carotid arteries were double ligated with surgical silk sutures. The neck wound was sutured. Sham-operated group rats were treated similarly, except that the common carotid arteries were not occluded. During the surgical procedure, the rats were placed on a heating pad to maintain body temperature at 37.5 ± 0.5°C and remained on it until they recovered from anesthesia. The survival rate was 85.19% after BCCAO (with a total of 115 rats) and 91.11% in the sham group (with a total of 41 rats).

### Pharmacological Manipulation

Capsaicin (Alomone Labs) was dissolved in a vehicle containing sterile 0.9% saline (ST341, Beyotime), dimethyl sulfoxide (DMSO, ST038, Beyotime), and Tween 80 (80/10/10%, V/V) (Chen et al., 2017; Hakimizadeh et al., 2017). The vehicle or drug solution (1 mg/kg) was injected intraperitoneally (i.p.), and immediately after BCCAO daily throughout the experiment. The dose of capsaicin used in this study was chosen based on previous studies (Chen et al., 2017). All other chemicals were of analytical grade and procured from local suppliers shown in the Supplementary Material. Animals in each group were given intervention in the same environment.

### Behavioral Analyses

**FIGURE 1:**
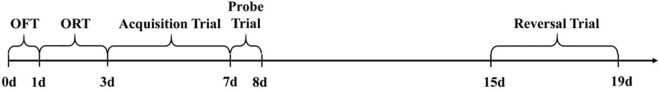
Time-line of the behavioral analyses.

We conducted behavioral tests, such as the open field test (OFT), object recognition test (ORT) and Morris water maze (MWM), to assess the spatial learning and memory abilities of rats (Figure 1). All behavioral tests were performed from 09:00 am to 14:00 pm in a sound-attenuated, air-regulated and constant light intensity experimental room to minimize the effects of circadian rhythms and glucocorticoids (de Quervain et al., 1998; Gerstner and Yin, 2010). One investigator, who was blinded to the group allocations, performed all behavioral tests to prevent interobserver variability caused by differences in the handling of rats.

#### Open Field Test

The OFT is a useful and simple test assessing the activity and general behavior (including locomotor activity/spontaneous behavior and exploration, memory, or anxiety) of rats (Deacon, 2006; Xu et al., 2020). As described in our previous study, briefly, a rat was placed into a corner square of the open field, facing the corner (He et al., 2019). The animal was observed for 5 min and the number of rearing behaviors (both front paws off the ground, with front paws against a wall, or with the animal freestanding) was recorded. The average speed, the total distance traveled in the OFT, the activity in the OFT and the distance traveled in the central area of the chamber were recorded by video analysis, and any feces were counted by hand. The apparatus was cleaned with 75% alcohol before the next rat performed the test.

#### Object Recognition Test

The ORT was performed using the procedure previously described (Bevins and Besheer, 2006; He et al., 2019). The ORT chamber was a black polyvinyl chloride (PVC) arena (40 × 40 × 45 cm), and the objects used were easy-to-clean plastic materials of similar sizes. Each animal was allowed a 2-day habituation session in the apparatus for 5 min, which was substituted by the OFT. Briefly, the ORT was divided into two parts. During the acquisition phase, two identical objects were placed in the right and left corners of the box. The rat was placed in the experimental box, allowed to explore freely for 5 min, and then returned back to its home cage. Ninety minutes later, the objects were replaced with another set of objects consisting of a familiar object and a novel object. The animal was returned to the arena and allowed to explore the objects for 5 min during the recognition phase. The time spent interacting with each object and the overall time exploring the objects (total exploration time), both the familiar and the new, was measured by video analysis. The discrimination index (DI) was calculated as follows: DI = N/(N + F) × 100%, where N represents the time spent interacting with the novel object and F represents the time spent contacting the familiar object. We calculated DI to evaluate the animal’s object-distinguishing memory [the test is regarded as an assessment of short-term recognition memory involving hippocampal activity (Kesner and Rolls, 2015)]. Animals able to discriminate between the new object and the old object should have a DI greater than 50% (Fréchou et al., 2019).

#### Morris Water Maze

The MWM test was performed using the protocol described in our previous studies (Wang et al., 2016; He et al., 2019). The device consisted of a painted black circular pool (diameter 120 cm) filled with water (temperature approximately 23 ± 1°C, 30 cm depth) in which a black escape platform (diameter 10 cm, 2 cm beneath the water surface) was hidden. The maze was divided into four quadrants and 4 points conceptually. Four colored clues to guide the animal were placed inside the pool, which were maintained unchanged throughout the experimental period. The behavior of the rats in the pool was recorded by a video camera positioned over the pool, which was connected to a computer-based image analyzer MWM tracking system (MS-1, Chengdu Instrument Factory, Chengdu, China). During the entire experiment, the lighting of the testing room indirectly illuminated the pool, and the environment (e.g., experimenter, work table, door, and pipes) was kept consistent.

Rats were separately trained to locate the hidden platform for acquisition training trials, in four trials per day over five consecutive days with a training interval of 15 min to assess spatial learning and reference memory. For each trial, a rat was randomly placed in the pool at one of the four points (according to the random number table) as the start position and allowed to swim freely for 1 min until it found and climbed onto the hidden platform (placed in the southwest of the maze). After successfully reaching the hidden platform, the rat was allowed to remain on the platform for 10 s. The time the rats first climbed onto the platform was recorded. If unsuccessful, the experimenter guided the rat to swim onto the platform and remain there for 10 s, and counted the time as 60 s. On day 6, the platform was removed in the probe trial to assess spatial memory. Similar procedures were performed for reversal trials to detect working memory, only different in a training interval of 5 min, with continuous training for 4 days. The time the rats first passed the platform, time spent in the target quadrant and frequency of passing the platform location were recorded.

### Histomorphological and Biochemical Analyses

After behavioral experiments, the experimental procedures were performed, including sacrifice of the animal, skull gaffing and hippocampus removal, for histomorphological and biochemical analyses.

#### Brain Tissue Collection and Analyses

All animals were anesthetized deeply with pentobarbital sodium (80 mg/kg, i.p.), the chest was opened quickly to fully expose the heart, and the animals were perfused transcardially with 400 ml of 4°C saline. Brains were extracted rapidly and dissected through the midsagittal plane. Half of the right hemisphere was fixed with 4% paraformaldehyde (AR1069, Boster) for 24 h and embedded in optimum cutting temperature (OCT) compound (SAKURA, Pennsylvania, United States) after concentration gradient dehydration for cryosection, which was stored at −80°C until analysis. The hippocampus in the left hemisphere was dissected immediately, and half of the hippocampus including the CA1 area, was fixed with 2.5% glutaraldehyde (P1127, Solarbio, Beijing, China) and embedded in epoxy resin for the preparation of ultrathin sections. The other hemisphere was snap-frozen in liquid nitrogen and stored at −80°C until analysis. All operations were performed on ice.

#### Western Blotting and Immunoblotting

Proteins were extracted with a total protein extraction kit (BC3711, Solarbio, Beijing, China) according to the manufacturer’s protocol, and we detected the protein level using a BCA protein assay kit (P0012S, Beyotime, Shanghai, China). Samples were prepared in 4x sample loading buffer (P1015, Solarbio, Beijing, China) and heated to 100°C for 5 min. Equal amounts of protein sample (60 μg) were separated by SDS-PAGE on a 10% polyacrylamide gel using a Mini-PROTEAN 3 gel electrophoresis system and Transblot system (Bio-Rad, California, United States), and the proteins were transferred to a PVDF membrane (Millipore, Massachusetts, United States). The immunoblots were then blocked by incubating them with 5% (W/V) non-fat dry milk and 0.1% (W/V) Tween-20 (T8220, Solarbio) in Tris-buffered saline (TBS, T1080, Solarbio) for 2 h at room temperature and then probed with a primary antibody (anti-mitofusin2 antibody, ab56889, Abcam, 1:500; or anti-GAPDH antibody, ab8245, Abcam, 1:1000) diluted in blocking solution and gently shaken overnight at 4°C (Paillusson et al., 2017). After washes with blocking solution (0.1% TBST, three times, 10 min each), the membranes were incubated with horseradish peroxidase-conjugated goat anti-mouse IgG (SA00001-1, Proteintech, 1:2000) for 2 h at room temperature. Images were acquired on BioSpectrum Imaging System. Signals on immunoblots were quantified using ImageJ software (National Institutes of Health, Bethesda, Maryland, United States), with MFN2 values normalized to GAPDH.

#### Light Microscopy

Sections of brain tissue (at 10 μm-thickness) were acquired using a Leica freezing microtome, hippocampus was taken continuously from the lateral geniculate body level. After washed with PBS (P1010, Solarbio), the samples were blocked with 10% goat serum/PBS plus 0.5% Triton X-100 for 1 h and then incubated with primary antibodies [anti-outer mitochondrial membrane protein-20 (TOMM20) antibody to label mitochondria, ab78547, Abcam, 1:200/anti-protein disulfide isomerase (PDI) antibody to label ER, ab2792, Abcam, 1:250] diluted in blocking solution. Next, samples were washed with PBS and incubated with goat anti-rabbit/goat anti-mouse secondary antibodies conjugated to Alexa Fluor 488/Alexa Fluor 594 (Invitrogen, California, United States, 1:1000) for 2 h. After washed with PBS, the samples were counterstained with 4′,6-diamidino-2-phenylindole (DAPI, blue) to label the nuclei. Following the final washes, images were captured using an Olympus microscope (IX81, Japan) and Nikon confocal microscope (A1R +, Japan). Colocalized pixels were quantified using ImageJ software to analyze the ER-mitochondria interactions.

#### Electron Microscopy

Small pieces of brain tissue (approximately 1 mm^3^ cubes) were fixed with 2.5% glutaraldehyde for at least 2 h at 4°C. After washed with 0.1 M sodium cacodylate buffer, the tissues were postfixed with 1% osmium tetroxide (OsO4), and dehydration in a concentration gradient was subsequently performed. Then, the samples were embedded in epoxy resin (Epon 812). Ultrathin sections (approximately 90 nm) were generated after localization using a Reichert-Jung Ultracut E Ultramicrotome (Lab X), placed on copper grids, stained with lead citrate and examined under a transmission electron microscope (TEM, Hitachi, H600-IV, Japan). The mitochondrial-associated ER membranes were delineated using the freehand tool. An investigator quantified the images in a blinded manner. We calculated the circumference of mitochondrial surface closely associated with ER (<30 nm). For the normalization of MAMs, we examined the circumference of mitochondrial perimeter to calculated the proportions of the MAMs (Arruda et al., 2014; Paillusson et al., 2017). The images were randomly selected without prior knowledge of groups for analyses using Image-Pro Plus 6.0 software.

### Statistical Analysis

An experimenter blind to the group allocations conducted all data collection and analysis procedures. All data are presented as means ± SD. Statistical analyses were performed with SPSS software (version 18.0 for Windows, SPSS Inc., New York, NY, United States), and graphs were generated using GraphPad Prism software (La Jolla, CA, United States). Two-way repeated-measures analysis of variance (ANOVA) followed by Fisher’s least significant difference (LSD) *post hoc* test were used to analyze the data from the MWM acquisition and reversal trials, and unpaired *t*-tests were used to analyze the number of crossings of the platform location and time spent in target quadrant in the probe trials of the MWM. Mauchly’s test of sphericity followed by the Greenhouse–Geisser (G–G) test were used to analyze the interaction effects. The Shapiro–Wilk test was used for the normality test of studentized residuals. One-way ANOVA was used to examine the effect of surgery and capsaicin on other behavioral experimental data, followed by Fisher’s LSD *post hoc* test. All comparisons of immunohistochemical, immunoblotting and morphological data among groups were performed using one-way ANOVA followed by Fisher’s LSD *post hoc* test. Data were tested for normality using the Shapiro–Wilk test. A probability of less than 0.05 was considered significant.

## Results

### Loosened Endoplasmic Reticulum-Mitochondria Contacts in the Hippocampal CA1 Region of Chronic Cerebral Hypoperfusion Rats

**FIGURE 2:**
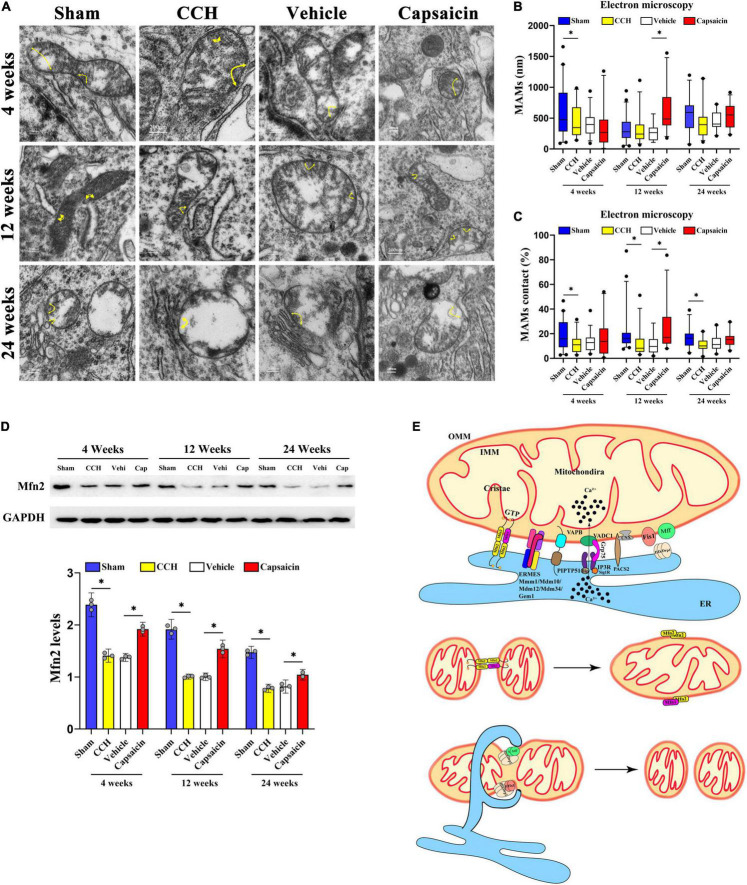
Capsaicin rescues loosened ER-mitochondria contacts in the hippocampal CA1 region of CCH rats. **(A)** Representative electron micrographs of ER-mitochondria contacts at each time point in each group. Yellow arrowheads with loops show regions of contact. Scale bar = 200 nm (20000x). **(B)** Boxplot showing the length of the mitochondrial surface closely contacted with the ER (<30 nm) at different time points. **(C)** The proportion of ER-mitochondrial contacts relative to the mitochondrial circumference. **(D)** Scatterplot showing the relative MFN2 levels. **(E)** Schematic drawing of MAMs. The components mainly included the MFN2 homodimer; the MFN1/2 heterodimer; endoplasmic reticulum mitochondria encounter structure (ERMES) composed of Mdm10/34/12, Mmm1 and Gem1; and VAMP-associated protein B (VAPB) interacting with PTPIP51. Data were analyzed by one-way ANOVA followed by the *post hoc* LSD test (data obtained from 100 to 124 mitochondria). Data are presented as the mean ± SD. *P < 0.05.

A reduction of apposition of ER associated with mitochondria has been reported in PD (Guardia-Laguarta et al., 2014). Alteration of the physical association between the ER and mitochondria has been reported to be involved in AD (Eysert et al., 2020). The processes affected by the MAMs are widely implicated in cognitive impairment (Liu and Zhu, 2017). However, no data regarding ER-mitochondria contacts in CCH rats have been published. We utilized TEM to monitor ER and mitochondrial morphology and physical associations and to determine whether CCH disrupts MAMs. We observed substantial damage to the mitochondrial ultrastructure in the hippocampal CA1 area of the CCH and vehicle control groups, especially in 24 weeks, as evidenced by altered mitochondrial shape and cristae, as well as mitochondrial vacuolation (Figure 2A), consistent with previous studies (He et al., 2019; Wang et al., 2020b). Mitochondrial vacuolation and other structural alterations, including those in cristae, were consistent with mitochondrial degeneration (Choi et al., 2014).

We then analyzed the length of domains of the mitochondrial surface closely connected to the ER (<30 nm) (Arruda et al., 2014; Paillusson et al., 2017). The length of ER-mitochondria associations was decreased only at 4 weeks after BCCAO surgery compared to sham group (Figure 2B, 4 weeks: *P* = 0.038; 12 weeks: *P* = 0.869; 24 weeks: *P* = 0.115). Interestingly, further analysis indicated that the proportion of the ER-mitochondria associations relative to the mitochondrial circumference was obviously decreased in the CCH group at all time points compared to sham group (Figure 2C, 4 weeks: *P* = 0.013; 12 weeks: *P* = 0.013; 24 weeks: *P* = 0.003). These methods have been applied previously (Stoica et al., 2014, 2016; Filadi et al., 2015; Paillusson et al., 2017; Leal et al., 2018). Based on these data, CCH appeared to significantly reduce the ER-mitochondria contacts, particularly a reduced contact area in proportion but not distance. In addition, we quantified MFN2 expression in the CA1 region. The MAMs contacts depend on MFN2 (de Brito and Scorrano, 2008), and the CCH group with loosened ER-mitochondria contacts showed lower MFN2 expression (Figure 2D, 4 weeks: *P* < 0.0001; 12 weeks: *P* < 0.0001; 24 weeks: *P* < 0.0001).

### Capsaicin Rescues Loosened Mitochondria-Associated Endoplasmic Reticulum Membranes Induced by Chronic Cerebral Hypoperfusion

Capsaicin has been reported to alleviate mitochondrial dysfunction accompanied by reduced MAM formation by activating TRPV1 (Wei et al., 2020) and has been proposed to upregulate MFN2 (Su et al., 2017). We then quantified MAMs in the capsaicin-treated group. The length of ER-mitochondria contacts was obviously increased in the capsaicin-treated groups compared to the vehicle control group, although the differences were not significant except in the 12-week group (Figure 2B, 4 weeks: *P* = 0.402; 12 weeks: *P* < 0.0001; 24 weeks: *P* = 0.276). A similar experimental result was obtained for the proportion of ER-mitochondrial contacts (Figure 2C, 4 weeks: *P* = 0.236; 12 weeks: *P* = 0.001; 24 weeks: *P* = 0.137), suggesting that the ultrastructure appeared to change after 12-week capsaicin treatment. Moreover, we detected no difference between the CCH and vehicle control groups (Figure 2B, 4 weeks: *P* = 0.892; 12 weeks: *P* = 0.662; 24 weeks: *P* = 0.945; Figure 2C, 4 weeks: *P* = 0.628; 12 weeks: *P* = 0.968; 24 weeks: *P* = 0.500). Compared with the vehicle control rats, MFN2 was expressed at higher levels in the CA1 region of the hippocampus in capsaicin-treated rats (Figure 2D, 4 weeks: *P* < 0.0001; 12 weeks: *P* < 0.0001; 24 weeks: *P* < 0.0001). No differences in the MFN2 level were detected in the CCH and vehicle control groups at any time point (Figure 2D, 4 weeks: *P* = 0.515; 12 weeks: *P* = 0.932; 24 weeks: *P* = 0.366). All these data indicate the alterations in loosened MAMs in CCH (Figure 2E) after capsaicin treatment.

### Capsaicin Rescues Low Endoplasmic Reticulum-Mitochondria Colocalization in Chronic Cerebral Hypoperfusion Rats

**FIGURE 3:**
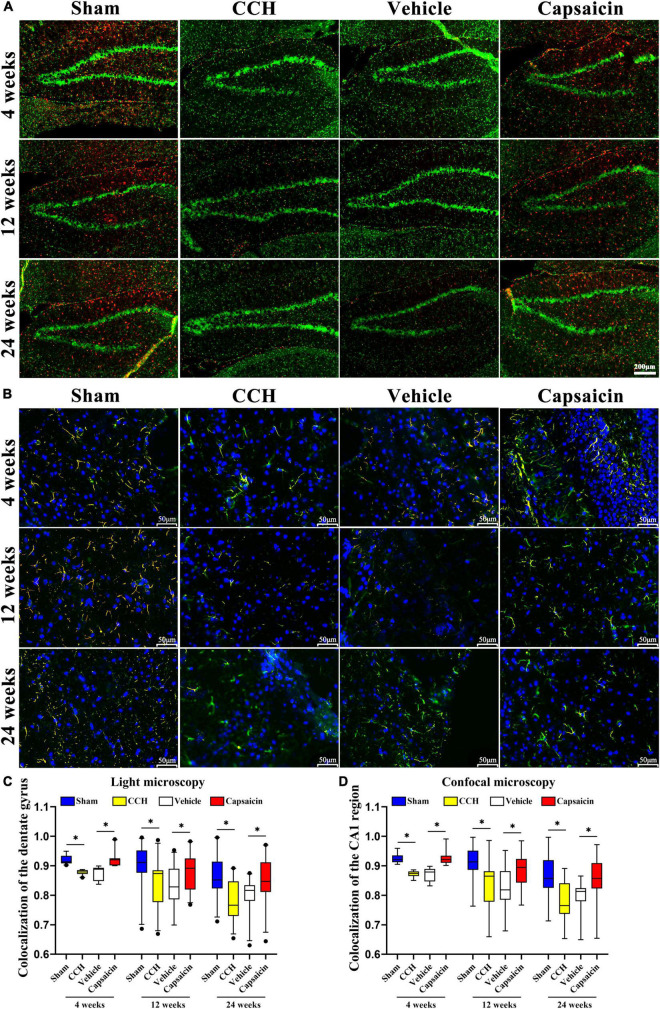
The reduced ER-mitochondria colocalization in CCH rats is reversed by capsaicin. **(A)** Representative light micrographs of ER-mitochondria contacts in the dentate gyrus of the hippocampus at each time point in each group. MAMs were measured by determining ER and mitochondria colocalization using ImageJ through immunostaining for PDI and TOM20 to label the ER (red) and mitochondria (green), respectively, and merged signals indicate colocalization (orange). Scale bar is 200 μm (100x). **(B)** Representative confocal images of ER-mitochondria contacts in the CA1 region of the hippocampus. Nuclei appear in blue. The scale bar represents 50 μm (200x). **(C)** Boxplot showing the colocalization values in the hippocampus (*n* = 22 – 29 per group). **(D)** Boxplot showing the colocalization values in the hippocampal CA1 region of different groups (*n* = 66 – 87 per group). Data are presented as the mean ± SD. *P < 0.05. Data were analyzed by one-way ANOVA followed by the *post hoc* LSD test.

**FIGURE 4:**
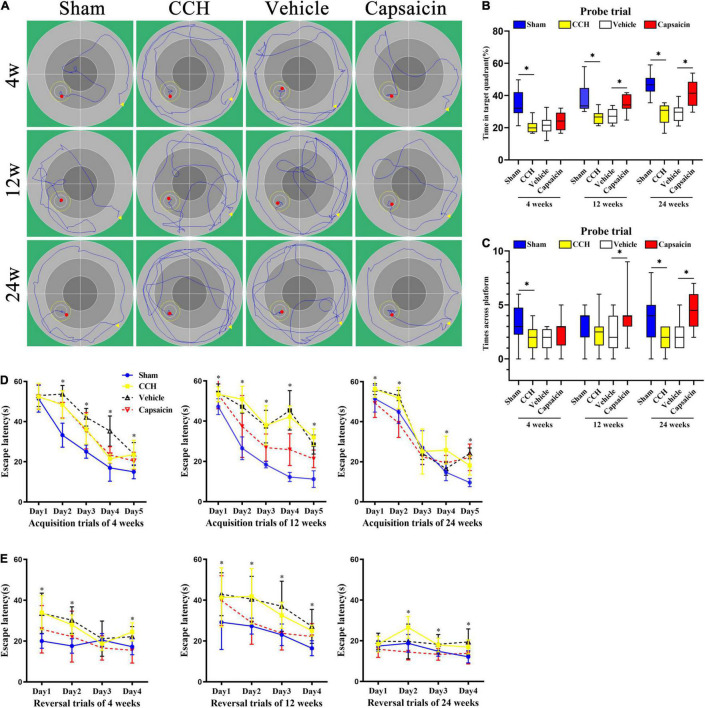
Capsaicin reverses CCH-induced spatial learning and memory impairments in the MWM. **(A)** Representative activity traces of the MWM (*n* = 12 rats/group). **(B)** Spatial reference memory was analyzed using the probe trial, boxplot showing the percentage of time spent in the platform quadrant. **(C)** The number times rats crossed the original platform location. **(D)** Spatial learning was assessed by acquisition trials of the MWM. CCH rats (yellow squares), sham-operated rats (blue circles), capsaicin-treated rats (red inverted triangles) and vehicle-control rats (black triangles) were tested at 4, 12, and 24 weeks after BCCAO. Escape latency to the hidden platform during acquisition trials of the MWM at (left panel) 4 weeks, (middle panel) 12 weeks, and (right panel) 24 weeks. **(E)** Spatial working memory was impaired in CCH rats during reversal trials at (left panel) 4 weeks, (middle panel) 12 weeks, and (right panel) 24 weeks, and capsaicin-treated rats had significantly shorter escape latencies than vehicle control rats at (left panel) 4 weeks, (middle panel) 12 weeks and (right panel) 24 weeks. Data are presented as the mean ± SD. *P < 0.05. Data were analyzed by two-way ANOVA followed by the *post hoc* LSD test, and unpaired *t*-tests were used to analyze probe trials’ number of crossings the platform and time spent in target quadrant.

Since ER-mitochondria interactions produced particularly striking electron micrographs, we reasoned that these phenotypes would be clearly discernible using light microscopy. We further characterized the effects of ER-mitochondria colocalization at different levels of the hippocampus, utilizing an immunofluorescence histochemical double-staining method and observed them with fluorescence microscopy and confocal microscopy (Stoica et al., 2014). The ER was labeled with a PDI antibody, and mitochondria were labeled with a TOMM20 antibody (mitochondrial outer membrane), colocalized pixels were quantified by using ImageJ. As expected, colocalization of ER and mitochondria was significantly decreased in the CCH groups compared to the sham groups in the hippocampus. We showed the colocalization of the dentate gyrus region in Figures 3A,C (4 weeks: *P* < 0.0001; 12 weeks: *P* = 0.004; 24 weeks: *P* < 0.0001), and the CA1 area in Figures 3B,D (4 weeks: *P* < 0.0001; 12 weeks: *P* < 0.0001; 24 weeks: *P* < 0.0001). Interestingly, we detected a significant change in ER-mitochondria colocalization in the dentate gyrus of the hippocampus, after the capsaicin intervention compared to the vehicle-control groups (Figures 3A,C, 4 weeks: *P* < 0.0001; 12 weeks: *P* = 0.034; 24 weeks: *P* = 0.011). A similar phenomenon was observed in the CA1 region (Figures 3B,D, 4 weeks: *P* < 0.0001; 12 weeks: *P* < 0.0001; 24 weeks: *P* < 0.0001). We observed no significant difference in the CCH and vehicle control groups (Figures 3A,C, 4 weeks: *P* = 0.496; 12 weeks: *P* = 0.827; 24 weeks: *P* = 0.368; Figures 3B,D, 4 weeks: *P* = 0.345; 12 weeks: *P* = 0.734; 24 weeks: *P* = 0.132).

### Spatial Learning and Memory Are Impaired in Chronic Cerebral Hypoperfusion Rats

The hippocampus plays an indispensable role in spatial and non-spatial memory (Morris et al., 1982; Sawangjit et al., 2018). We examined spatial learning and memory using the MWM. First, rats performed cued training with a marked goal to become familiar with the facility and to exclude possible motor and/or sensory deficits. A significant operation × trial interaction was observed for escape latency after BCCAO [4 weeks, *F*_*operation* × *trial*_(4,44) = 5.020, P = 0.002; 12 weeks, *F*_*operation* × *trial*_ (4,44) = 15.243, *P* < 0.0001; 24 weeks, *F*_*operation* × *trial*_(4,44) = 3.628, *P* = 0.012, respectively]. The *post hoc* analysis indicated a decreasing trend for latency across trials [Figure 4D, 4 weeks: *F*_*trial*_ (4,44) = 162.503, *P* < 0.0001; 12 weeks: *F*_*trial*_(4,44) = 141.706, *P* < 0.0001; 24 weeks: *F*_*trial*_ (4,44) = 180.477, *P* < 0.0001]. A statistically significant operation effect on escape latency suggested a robust recognition memory deficit in CCH rats, as the CCH rats took longer to reach the submerged platform than the rats in the sham-operated group [Figure 4D, 4 weeks, *F*_*operation*_(1,11) = 37.606, *P* < 0.0001; 12 weeks, *F*_*operation*_(1,11) = 488.967, *P* < 0.0001; 24 weeks, *F*_*operation*_(1,11) = 19.364, *P* = 0.001]. Together, these findings suggested that during a 5-day acquisition trial, all groups were able to finish the trial and learn the task at different time points, with a tendency toward a decreased escape latency to find the hidden platform. Additionally, spatial learning performance was impaired in CCH rats at all time points. Differences in escape latency were not due to swimming speed, which showed minimal differences across the CCH and sham-operated groups at all time points [Supplementary Figure 1A, 4 weeks: *F*_*operation*_(1,11) = 0.005, *P* = 0.944; 12 weeks: *F*_*operation*_ (1,11) = 0.101, *P* = 0.757; 24 weeks: *F*_*operation*_ (1,11) = 4.280, *P* = 0.063, respectively].

Spatial reference memory was assessed in the probe trial. In summary, the probe trial results suggested that sham-operated rats displayed a significant preference for the target quadrant, as shown by the increased percentage of time spent in the platform quadrant [Figure 4B, 4 weeks: *t*(22) = 4.470, *P* < 0.0001; 12 weeks: *t*(22) = 3.840, *P* = 0.001; 24 weeks: *t*(22) = 6.767, *P* < 0.0001, respectively]. The sham-operated group showed better performance in finding the goal location, as indicated by the increased number of crossings in the platform area at 4 and 24 weeks after BCCAO. At 12 weeks after surgery, CCH rats showed the same pattern as the other time points, but the difference failed to reach significance [Figure 4C, 4 weeks: *t*(22) = 2.351, *P* = 0.028; 12 weeks: *t*(22) = 0.930, *P* = 0.362; 24 weeks: *t*(22) = 2.434, *P* = 0.024, respectively]. Swimming speed did not differ significantly between the groups at different time points [Supplementary Figure 1B, 4 weeks: *t*(22) = 0.193, *P* = 0.849; 12 weeks: *t*(22) = 1.037, *P* = 0.311; 24 weeks: *t*(22) = 0.072, *P* = 0.944]. Typical swimming path diagrams are shown in Figure 4A.

Spatial working memory was assessed in reversal trials, and CCH rats took more time to find the new goal position than sham-operated rats [Figure 4E, *F*_*operation*_(1,11) = 55.170, *P* < 0.0001; *F*_*operation*_(1,11) = 17.772, *P* = 0.001; *F*_*operation*_(1,11) = 33.605, *P* < 0.0001]. No significant difference in swimming speed was observed [Supplementary Figure 1C, 4 weeks: *F*_*operation*_(1,11) = 1.367, *P* = 0.267; 12 weeks: *F*_*operation*_(1,11) = 2.193, *P* = 0.167; 24 weeks: *F*_*operation*_(1,11) = 0.516, *P* = 0.488].

### Capsaicin Rescues Spatial Learning and Memory Impairments Induced by Chronic Cerebral Hypoperfusion

Capsaicin has been proven to prevent hippocampal synaptic plasticity and spatial memory retrieval (Li et al., 2008) and seems to exert a neuroprotective effect on cognitive function (Pegorini et al., 2005; Avraham et al., 2009; Du et al., 2020). Based on these previous findings, we hypothesized that capsaicin would rescue cognitive impairments induced by CCH. During the acquisition of spatial learning, all groups showed a significantly (progressively) decreased escape latency to find the target platform in the capsaicin-treated groups and vehicle control groups [Figure 4D, 4 weeks: *F*_*trial*_(4,44) = 93.194, *P* < 0.0001; 12 weeks: *F*_*trial*_(4,44) = 39.968, *P* < 0.0001; 24 weeks: *F*_*trial*_(4,44) = 231.773, *P* < 0.0001]. A statistically significant treatment × time interaction was observed [4 weeks: *F*_*treatment* × *trial*_(4,44) = 4.273, *P* = 0.005; 12 weeks: *F*_*treatment* × *trial*_(4,44) = 4.253, *P* = 0.005; 24 weeks: *F*_*treatment* × *trial*_(4,44) = 10.535, *P* < 0.0001], and the *post hoc* analysis revealed that capsaicin-treated rats found the platform more quickly than vehicle control rats, as shown by a shorter escape latency [Figure 4E, 4 weeks: *F*_*treatment*_(1,11) = 16.539, *P* = 0.002; 12 weeks: *F*_*treatment*_(1,11) = 72.562, *P* < 0.0001; 24 weeks: *F*_*treatment*_(1,11) = 58.675, *P* < 0.0001]. Swimming speed showed no confounding effects on motor impairments and did not alter the escape latency [Supplementary Figure 1A, 4 weeks: *F*_*treatment*_(1,11) = 0.753, *P* = 0.404; 12 weeks: *F*_*treatment*_(1,11) = 0.003, *P* = 0.954; 24 weeks: *F*_*treatment*_(1,11) = 0.043, *P* = 0.840].

In the spatial reference memory test performed on day 6 by removing the original platform, significant differences in the percentage of time spent in the target quadrant (excluding 4 weeks after BCCAO treated by capsaicin compared to vehicle control groups) were found between groups [Figure 4B, 4 weeks: *t*(22) = 0.950, *P* = 0.352; 12 weeks: *t*(22) = 3.918, *P* = 0.001; 24 weeks: *t*(22) = 4.485, *P* < 0.0001, respectively]. Additionally, capsaicin-treated rats swam across the platform area more times than rats in the vehicle control groups, except at 4 weeks after BCCAO, although they showed a similar trend [Figure 4C, 4 weeks: *t*(22) = 1.406, *P* = 0.174; 12 weeks: *t*(22) = 2.145, *P* = 0.043, respectively; 24 weeks: *t*(22) = 3.354, *P* = 0.003, respectively]. No significant difference in swimming speed was observed at any time point [Supplementary Figure 1B, 4 weeks: *t*(22) = 0.279, *P* = 0.783; 12 weeks: *t*(22) = 0.001, *P* = 0.999; 24 weeks: *t*(22) = 0.088, *P* = 0.931, respectively].

Regarding spatial working memory, vehicle control rats took significantly longer to find the new position than capsaicin-treated rats in the reversal trials [Figure 4E, 4 weeks: *F*_*treatment*_(1,11) = 52.433, *P* < 0.0001; 12 weeks: *F*_*treatment*_(1,11) = 28.782; 24 weeks: *F*_*treatment*_(1,11) = 20.948, *P* = 0.001]. The differences were not attributed to the swimming speed [Supplementary Figure 1C, 4 weeks: *F*_*treatment*_(1,11) = 1.909, *P* = 0.194; 12 weeks: *F*_*treatment*_(1,11) = 0.089, *P* = 0.771; 24 weeks: *F*_*operation*_(1,11) = 0.085, *P* = 0.776]. Interaction effects were not observed among vehicle control and capsaicin-treated comparisons in reversal trials. The escape latency of the vehicle control group did not differ significantly from that of the CCH group [Supplementary Figure 1C, 4 weeks: *F*_*treatment*_(1,11) = 1.909, *P* = 0.194; 12 weeks: *F*_*treatment*_(1,11) = 0.089, *P* = 0.771; 24 weeks: *F*_*treatment*_(1,11) = 0.085, *P* = 0.776].

### Capsaicin Relieves the Non-associative Learning or Habituation Impairment and Anxiety-Like Behavior Caused by Chronic Cerebral Hypoperfusion

**FIGURE 5:**
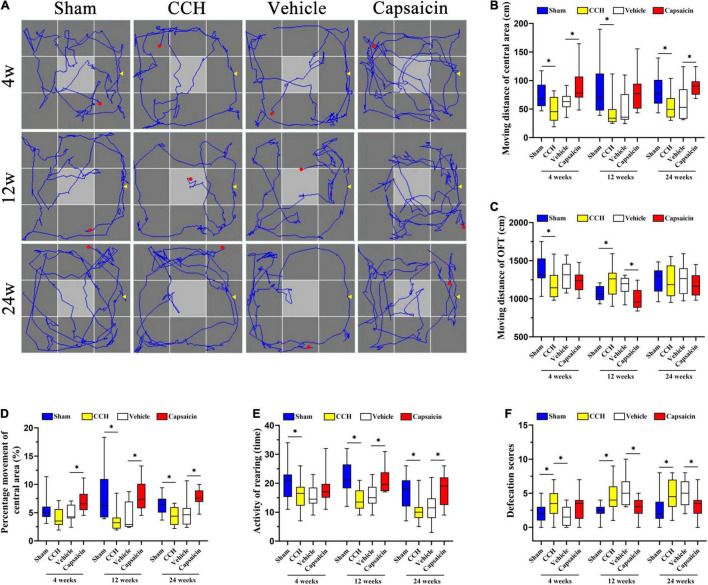
Capsaicin relieves the changes in non-associative learning and anxiety-like behavior caused by CCH. **(A)** The rats’ typical performance in the OFT. **(B)** The distance traveled in the central area (Dc) (*n* = 12). **(C)** The total distance (Dt) traveled in the OFT. **(D)** The percentage of distance traveled in the central area was higher in the sham group and capsaicin-treated group. **(E)** The rearing activity in the OFT. **(F)** Defecation scores in the OFT. Data are presented as the mean ± SD. *P < 0.05. Data were analyzed by one-way ANOVA followed by the *post hoc* LSD test.

We have previously reported that CCH rats have significant cognitive deficits, including non-associative learning or habituation impairment and obvious anxiety-like behavior (Wang et al., 2016; He et al., 2019). TRPV1-deficient mice showed reduced anxiety, conditioned fear memory, and LTP in the hippocampal CA1 area (Marsch et al., 2007). Here, we used the OFT to assess the locomotor activity or exploratory and anxiety-like behaviors of rats (Deacon, 2006; Johnson et al., 2018; Xu et al., 2020) and determined the typical path of the rats in the OFT (Figure 5A). As shown in Figure 5B, the distance traveled in the central area (Dc) by CCH rats was obviously longer than that of sham-operated rats. The result was significant in the *post hoc* analysis (*n* = 12, 4 weeks: *P* = 0.010; 12 weeks: *P* = 0.008; 24 weeks: *P* = 0.010). Regarding the total distance traveled (Dt), the CCH and sham-operated groups produced similar results at 24 weeks (Figure 5C, 4 weeks: *P* = 0.013; 12 weeks: *P* = 0.008; 24 weeks: *P* = 0.824). We further analyzed the percentage of distance traveled in the central area (i.e., Dc/Dt × 100%) to evaluate the related cognitive and emotional deficits in CCH rats. Interestingly, the percentage of distance traveled in the central area was significantly different between CCH and sham rats, except at 4 weeks (Figure 5D, 4 weeks: *P* = 0.053; 12 weeks: *P* = 0.002; 24 weeks: *P* = 0.009). In addition, the CCH and sham groups showed no differences in the average speed or the time spent in the central area, as illustrated in Supplementary Figures 1D,E (average speed: 4 weeks: *P* = 0.750; 12 weeks: *P* = 0.989; 24 weeks: *P* = 0.939; time of central area: 4 weeks: *P* = 0.179; 12 weeks: *P* = 0.193; 24 weeks: *P* = 0.136, respectively). Finally, we also analyzed rearing activity and defecation scores (Figure 5E, 4 weeks: *P* = 0.049; 12 weeks: *P* < 0.0001; 24 weeks: *P* = 0.004), and the results suggested that defecation scores were augmented in CCH group whereas locomotor activity was decreased (Figure 5F, 4 weeks: *P* = 0.033; 12 weeks: *P* = 0.006; 24 weeks: *P* = 0.022).

We simultaneously analyzed the effect of capsaicin on rats with CCH (*n* = 12). As depicted in Figure 5B, the distance traveled in the central area (Dc) by the capsaicin-treated group was increased at 4 weeks and 24 weeks, but not at 12 weeks (4 weeks: *P* < 0.0001; 12 weeks: *P* = 0.052; 24 weeks: *P* = 0.003). The total distance (Dt) showed the opposite trend (Figure 5C, 4 weeks: *P* = 0.348; 12 weeks: *P* < 0.0001; 24 weeks: *P* = 0.432). The percentage of distance traveled in the central area increased after treatment with capsaicin compared to the vehicle control group (Figure 5D, 4 weeks: *P* = 0.006; 12 weeks: *P* = 0.009; 24 weeks: *P* < 0.0001). The 4-week and 24-week groups showed significant differences in the times spent in the central area (Supplementary Figure 1E, 4 weeks: *P* = 0.001; 12 weeks: *P* = 0.141; 24 weeks: *P* = 0.004). However, the average speed of rats at all time points was no different (Supplementary Figure 1D, 4 weeks: *P* = 0.323; 12 weeks: *P* = 0.942; 24 weeks: *P* = 0.894). Finally, the results shown in Figure 5E (4 weeks: *P* = 0.171; 12 weeks: *P* = 0.006; 24 weeks: *P* = 0.006) suggest that the rearing activity was predominantly increased in the capsaicin-treated group compared to the vehicle control group at 12 and 24 weeks but not at 4 weeks. Additionally, we calculated the defecation scores, as shown in Figure 5F (4 weeks: *P* = 0.071; 12 weeks: *P* = 0.003; 24 weeks: *P* = 0.035) suggesting that defecation scores were increased after treated by capsaicin. The CCH and vehicle control groups showed no significantly differences except in defecation scores analysis (Figure 5B, 4 weeks: *P* = 0.158; 12 weeks: *P* = 0.733; 24 weeks: *P* = 0.582; Figure 5C, 4 weeks: *P* = 0.122; 12 weeks: *P* = 0.318; 24 weeks: *P* = 0.829; Figure 5D, 4 weeks: *P* = 0.280; 12 weeks: *P* = 0.611; 24 weeks: *P* = 0.606; Figure 5E, 4 weeks: *P* = 0.940; 12 weeks: *P* = 0.416; 24 weeks: *P* = 0.508; Figure 5F, 4 weeks: *P* = 0.026; 12 weeks: *P* = 0.380; 24 weeks: *P* = 0.694).

### Capsaicin Alleviates the Short-Term Recognition Memory Impairment Induced by Chronic Cerebral Hypoperfusion

**FIGURE 6:**
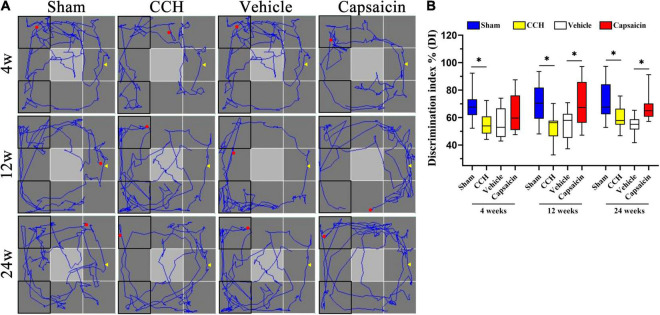
Capsaicin relieves the short-term recognition memory impairment induced by CCH. **(A)** The path diagram on the left shows the rats’ typical performance in the ORT. **(B)** The boxplot on the right shows the discrimination index (DI) in the ORT [the ratio between the time spent exploring the novel object (N) and the total exploration time (N + F)]. Data are presented as the mean ± SD. *P < 0.05. Data were analyzed by one-way ANOVA followed by the *post hoc* LSD test. N, the time spent interacting with the novel object; F, the time spent contacting the familiar object; N + F, the total exploration time.

Short-term recognition memory requires rapid storage and/or subsequent recall of intact memory. We used the ORT, which is widely used to evaluate non-spatial memory function after pharmacological challenges in mice and rats (Kassab et al., 2019; Berlin et al., 2020; Short et al., 2020), to further confirm the amelioration of recognition memory deficits induced by capsaicin. As shown in Figure 6B, one-way ANOVA revealed that CCH rats spent less time exploring the novel object than sham rats, yielding a discrimination index (DI) of 54.97 ± 7.98% at 4 weeks (*n* = 12), 53.09 ± 10.51% at 12 weeks (*n* = 12), and 59.57 ± 8.02% at 24 weeks (*n* = 12). In contrast, sham-operated rats spent more time exploring according to the DI, with values of 69.93 ± 12.24% (4 weeks, *P* = 0.002), 70.33 ± 14.85% (12 weeks, *P* = 0.003), 71.34 ± 14.13% (24 weeks, *P* = 0.006), respectively, consistent with the results of our earlier studies. This impairment was significantly alleviated in rats treated with capsaicin, since their DI was higher (62.41 ± 13.60%, 70.25 ± 16.70%, and 67.53 ± 9.69%, respectively) than the vehicle control group (55.20 ± 11.02%, 55.06 ± 10.48%, and 54.72 ± 6.17%, respectively), except for the DI at 4 weeks (which showed the same pattern but the difference did not achieve significance, 4 weeks, *P* = 0.129; 12 weeks, *P* = 0.008; 24 weeks, *P* = 0.003). As depicted in Supplementary Figures 1F,G, the four groups showed the same speed and spent approximately equal time exploring the two objects at each time point. We show the typical path in the ORT in Figure 6A.

## Discussion

The pathogenesis of cognitive impairment is complex. Vascular factors are known to contribute pathologically to almost all forms of dementia (Raz et al., 2016; Dichgans and Leys, 2017; Fouda et al., 2019) and are related to multiple pathophysiological processes, including white matter lesions (WMLs) (Thomas et al., 2015; Choi et al., 2016; Poh et al., 2020), blood-brain barrier breakdown (Liu et al., 2019), neurovascular unit dysregulation (Levit et al., 2020), neuronal loss (Mao et al., 2019), neuroinflammation (Poh et al., 2020), oxidative stress (Ozacmak et al., 2009), and mitochondrial dysfunction (Du et al., 2017; Su et al., 2018). The well-established rat model of CCH, which has been proven to induce long-lasting cognitive and behavioral impairments for 16–24 weeks, is commonly used to study VCI (Choi et al., 2011; Duncombe et al., 2017; Han et al., 2020; Wang et al., 2020b). It has been demonstrated that cerebral blood flow decreased immediately after BCCAO (Choy et al., 2006), accompanied with neuronal death and degeneration (Jing et al., 2015). However, CBF returned to the control level from 3 to 6 weeks, and even returned normal value at 6 months, while glial cells increased at 4 weeks after BCCAO (Choy et al., 2006; Jing et al., 2015). Decreased CBF could induce hypoperfusion in the brain, impair water channel and glymphatic function and trigger neuroinflammation, eventually lead to cognitive impairment (Yu and Venkat, 2019). The glymphatic system is a clearance and transportation system along perivascular channels via astrocytes-mediated interstitial fluid bulk flow (Iliff et al., 2012). Glymphatic dysfunction is reported implicated in neurodegeneration and VCI (Wang et al., 2017). AQP4 water channels, expression around blood vessels, facilitate waste clearance and regulate water homeostasis in the CNS, appear to be one of the essential targets for CNS disorders (Mestre and Hablitz, 2018; Salman et al., 2021a,b). In addition, it is suggested that CCH could induce a compensative mechanism to maintain CBF and neuronal degeneration. It is possibly involved in blood flow redistribution, angiogenesis, and recruitment of non-perfused capillaries (Yang et al., 2014). However, this limited compensation could not prevent cognitive decline.

Consistent with previous findings (Daulatzai, 2017; Wang et al., 2020b), our data suggest that CCH rats have early and long-lasting cognitive impairments, as manifested by decreases in both spatial learning and memory, as well as short-term recognition and non-associative learning impairments accompanied by anxiety-like emotion, in studies using the MWM, ORT, and OFT. Notably, in contrast to previous studies, our data on spatial reference memory at 12 weeks after BCCAO showed no significant differences, even we observed similar trends combining with previous data. We speculate that this might be because of the individual differences among the rats. We then enlarged sample size to decline the random variations of the effect, the result indicated that spatial reference memory of 12-week CCH group were obviously impaired (*P* < 0.0001).

As mentioned above, mitochondrial dysfunction might be a key factor contributing to cognitive decline in both humans and animal models (Kerr et al., 2017; Fernandez et al., 2019; Khacho and Harris, 2019; Wang W. et al., 2020). Our previous study documented a 4834 bp deletion in mtDNA and morphological changes in the mitochondria of CCH rats, which seem to have a strong association with cognitive behavioral deficits induced by CCH (He et al., 2019). Although the precise molecular mechanisms remain to be elucidated, we propose that mitochondrial dysfunction is roughly divided into genetic and non-genetic mitochondrial defects. On the one hand, the non-genetic defects of mitochondria include mitochondrial functional and structural defects. Firstly, in addition to generation of ATP, mitochondria encompass a wide array of functions, ranging from regulation of redox homeostasis and cell signaling (Beckervordersandforth et al., 2017; Ungvari et al., 2018; Khacho and Harris, 2019). Secondly, mitochondria are highly dynamic depending on mitochondrial fission and fusion. Disruption of mitochondrial dynamics might lead to neuronal death and cognitive impairment (Alexiou et al., 2017). Instead of isolated cellular organelles, mitochondria also communicate with other different structures through membrane contact sites (MCSs) to perform cell functions (Wu and Carvalho, 2018). Mitochondria-associated membranes (MAMs) are major subdomains, physically and biologically contact the ER with mitochondria. MAMs are involved in multiple essential molecular events, such as biogenesis, calcium and ROS signaling, lipid synthesis and transport, apoptosis, autophagy and mitophagy, mitochondrial dynamics and ER stress (ERS) (Gelmetti et al., 2017; Csordás et al., 2018; van Vliet and Agostinis, 2018). Perturbation of these membranes could result in neurological disorders (Veeresh et al., 2019). On the other hand, genetic mitochondrial defects are caused by impaired mitochondrial DNA (mtDNA) or nuclear DNA (Mastroeni et al., 2017). Many of these defects directly influence cognitive function, particularly the process of learning and memory (Kramer and Bressan, 2018; Khacho and Harris, 2019).

The structural and functional dysfunction of MAMs provides an explanation for the seemingly disparate features of neurodegenerative diseases (Krols et al., 2016; Liu and Zhu, 2017). It could reasonably be inferred that MAMs dysfunction might be a common pathway leading to cognitive impairment. The normal structure and function of ER-mitochondria connection is based on a set of proteins. For example, inositol-1,4,5-triphosphate receptors (IP3Rs) and voltage-dependent anion channel (VDAC1) could form bridging complexes to regulate Ca^2+^ transport. Glucose-regulated protein (GRP75) and ER resident protein 44 (ERp44) are crucial for ER stress, while calnexin (CNX) directly influences the process of protein folding. Such proteins include lipid synthesizing and trafficking enzymes [e.g., phosphatidylserine synthase (PSS1 and PSS2), fatty acid CoA ligase 4 (FACL4), serine active site containing 1 (SERAC1)] also participate in the formation of MAMs. Additionally, mitochondrial shaping-proteins [MFN1, MFN2 and sigma-1 receptor (Sig1R)] are involved in mitochondrial dynamics, and phosphofurin acidic cluster sorting protein-2 (PACS-2) along with Bcl-2 could control apoptosis and membrane traffic (Hedskog et al., 2013; Giorgi et al., 2015). The disruption of these proteins might lead to damage to ER-mitochondria associations. Since the ER-mitochondria contact regulates many cellular functions that are damaged in disease, MAMs provides a possible mechanism by which different neurodegenerative disease features might arise.

An interesting example is that α-synuclein binds to VAPB and disrupt the VAPB-PTPIP51 tethers resulting in looser ER-mitochondria contacts and the disruption of calcium exchange and ATP production (Paillusson et al., 2017). Studies have been showed that the presence of α-synuclein in the MAMs, which may explain the mitochondrial abnormalities of PD (Guardia-Laguarta et al., 2014). Recent findings suggested a potential relationship between MAMs and AD in studies of ER-mitochondrial apposition and MAMs’ function in cells from patients with AD, while γ-secretase activity is observed predominantly in MAMs (Area-Gomez and Schon, 2016). Presenilin 1 (PS1) and PS2 are important components of the γ-secretase complex, reported to be enriched in MAMs. PS2 have been found to enhance the MAMs by contact to MFN2 (Veeresh et al., 2019). Interestingly, several groups have reported that CCH alters the amyloid beta (Aβ) pathway and contribute the pathogenesis of AD by regulating the activity of β-secretase/γ-secretase (Choi et al., 2011; Cai et al., 2017) or inducing impaired Aβ transport and clearance (Ashok et al., 2016). Others researchers assessed 11 patients with decreased cerebral blood flow and suggested that longstanding cerebral hypoperfusion in humans does not cause Aβ accumulation or tau aggregation of (Hansson et al., 2018). Additionally, TDP-43 could disrupt ER-mitochondria associations by reducing the binding of VAPB-PTPIP51, which contribute to the pathological features of ALS/FTD (Stoica et al., 2014). CCH may cause a cascade of pathological changes including oxidative stress, neuroinflammation, mitochondrial dysfunction, abnormal lipid metabolism, calcium homeostasis disorder and neurotransmitter system dysfunction (Du et al., 2017). These pathophysiological changes have been reported to be common mechanisms of cognitive impairment (Krols et al., 2016; Paillusson et al., 2016; Liu and Zhu, 2017), which might be associated with MAMs, making MAMs a promising therapeutic target to restore cognitive disorders.

Thus, in this study, we investigated the subcellular compartment of MAMs in CCH. To the best of our knowledge, we are first to report that MAMs are altered in CCH. We focused on the CA1 subregion of the hippocampus, an essential area for spatial cognition and episodic memory, particularly for spatial representations and learning of object-place associations (Sawangjit et al., 2018; Sun and Jin, 2019). A direct analysis showed a statistically significant decrease in the distance of ER and mitochondrial contacts in only the 4-week groups using electron microscopy. We were surprised to find a decrease in the proportion of ER-mitochondria associations relative to the total mitochondrial circumference in CCH compared to the sham group at each time point, indicating that ER and mitochondrial tethering in CCH exhibited a looser state. Indeed, the same remarkable phenotype was detected using light microscopy, subsequently proving this point. This looser contact might be due to a decrease in MFN2 expression. It has been examined that MFN2 tethers the ER to mitochondria (de Brito and Scorrano, 2008). Our findings concerning MFN2 at the molecular level supported this finding, although MFN2 ablation increased ER-mitochondria coupling in another study (Filadi et al., 2015). Ischemia has a reducing effect on MFN2 expression (Rutkai et al., 2019). The loosened MAMs and the low expression levels of MFN2 detected in CCH rats might suggest a mechanism that induces cognitive impairment.

Notably, a paucity of pharmacological treatments that specifically prevent CCH modification or target MAMs are currently available. TRPV1 channel has been implicated in the maintenance of Ca^2+^ homeostasis, generation of ROS, inflammation and mitochondrial function, and targeting TRP channels has been under extensive investigation as a selective neuroprotective treatment of CNS disorders (Thapak et al., 2020). TRPV1 is highly expressed in microglial cells, and activation of TRPV1 by capsaicin directly affects microglia function, modulates synaptic neurotransmission (Marrone et al., 2017) including glutamatergic transmission in the dentate gyrus synapses (Chávez et al., 2014). Another type of TRPV, for example, TRPV4 is expressed in astrocytes, neurons of the circumventricular organs and in endothelium. Co-expression with AQPs such as AQP4 and AQP1 is sufficient for TRPV4 activation, which may cause cell swelling (Toft-Bertelsen and Križaj, 2017). APQs have been implicated in pathologies including brain edema of CNS disorders, which is associated with disrupted water and solute homeostasis (Markou et al., 2021; Salman et al., 2022). Pharmacological interventions targeting AQPs might pave the way for the treatment of CNS disorders. Moreover, TRPV4-mediated Ca^2+^ signals in astrocytes play vital roles in cerebral blood flow and neuronal metabolism (Dunn et al., 2013). Except for TRPV (vanilloid), the TRP superfamily of cation channels, including TRPC (canonical), TRPM (melastatin), TRPP (polycystin), TRPML (mucolipin), TRPA (ankyrin), and TRPN (exists only in invertebrates and fish) in the brain have been found to be involved in the pathology of neurological diseases (Wang R. et al., 2020).

In the present study, we explored the potential pharmacological effects of capsaicin, which might be associated with MAMs. Compared to the vehicle control group, the drug protected cognitive function, particularly at the 12 weeks after BCCAO in behavior tests, and no significant difference between the vehicle control and CCH groups was observed. This result is consistent with previous findings from multiple studies showing that capsaicin exerts powerful neuroprotective effects (Li et al., 2008; Jiang et al., 2013; Huang et al., 2017). This histopathological evaluation, in addition to the behavioral performance of capsaicin-treated rats, indicated a significant increase in ER-mitochondria colocalization and MFN2 expression for at least 4 weeks after BCCAO, particularly in the 24-week group. Nevertheless, 4 weeks after capsaicin treatment, the rearing activity, DI, number of crosses and percentage of time spent in the target platform location in the MWM did not show significant differences. Based on this result, ER-mitochondria interactions may be improved prior to behavioral performance, and a more prolonged treatment may be required to achieve neuroprotective effects. This effect might be associated with the ability of capsaicin to accelerate re-endothelialization by upregulating MFN2 through the activation of the TRPV1 receptor after vascular injury (Su et al., 2017). MFN2 overexpression ameliorates hypoxia-induced neuronal apoptosis (Peng et al., 2015), and downregulation of MFN2 might be an important mechanism of neuronal dysfunction or death after ischemia (Klacanova et al., 2019). MFN2 deletion induces age-dependent depletion, neurogenesis defects and cognitive decline through impaired mitochondrial dynamics, might be upstream of neural stem cell (NSC) self-renewal (Khacho et al., 2016). Capsaicin has been suggested to prevent memory dysfunction and hippocampal CA1 neuronal damage after global cerebral ischemia (Pegorini et al., 2005). It is hypothesized that the functional and histological protection after global ischemia is probably associated with reduced calcium influx and expression of *N*-methyl-D-aspartate (NMDA) receptor (Pegorini et al., 2005; Huang et al., 2017).

Thus, we speculated that MAMs may be a core alteration and a promising therapeutic target to cognitive impairment induced by cerebral hypoperfusion. In addition, MFN2 might be a potential biomarker to predict impaired cognitive function by indicating damage to MAMs in tissues. Therefore, further studies are required to explore the physiological and pathological roles of MAMs in the brain using advanced, miniaturized, real-time experimental approach, such as humanized self-organized models, organoids, 3D cultures and human organ-on-a-chip platforms (Kim et al., 2015; Papaspyropoulos et al., 2020; Salman et al., 2020). Multidisciplinary process is needed to support drug discovery in future research (Aldewachi and Al-Zidan, 2021; Salman and Al-Obaidi, 2021).

## Conclusion

In summary, our findings suggest that CCH may selectively induce structural changes of MAMs in the CA1 region of the hippocampus and subsequently result in cognitive impairment. Capsaicin improves learning and memory defects, rescues the expression of MFN2, and reverses the loss of ER-mitochondria coupling. MAMs may be a key link in this process and a promising therapeutic target. Therapeutic strategies designed to improve MAMs through the MFN2 pathway might be a promising approach to prevent cognitive impairment induced by CCH. Future studies are needed to provide insights into the precise molecular mechanisms.

## Data Availability Statement

The raw data supporting the conclusions of this article will be made available by the authors, without undue reservation.

## Ethics Statement

The animal study was reviewed and approved by the Animal Experiment Committee of The General Hospital of Western Theater Command.

## Author Contributions

MO and QW: conceptualization. MO, QZ, and JS: methodology and writing-original draft. JF, KY, and LL: investigation. YL: formal analysis. QW: resources, supervision, and funding acquisition. QW and ZW: writing-review and editing. JF and KY: visualization. All authors full access to all the data in the study and approved the final version of the manuscript.

## Conflict of Interest

The authors declare that the research was conducted in the absence of any commercial or financial relationships that could be construed as a potential conflict of interest.

## Publisher’s Note

All claims expressed in this article are solely those of the authors and do not necessarily represent those of their affiliated organizations, or those of the publisher, the editors and the reviewers. Any product that may be evaluated in this article, or claim that may be made by its manufacturer, is not guaranteed or endorsed by the publisher.

## Abbreviations

AD: Alzheimer’s disease
ALS: amyotrophic lateral sclerosis
AMPAR: GluA2-containing α-amino -3-hydroxy-5-methyl-4-isoxazolepropionic acid receptor
BBB: blood-brain barrier
BCCAO: bilateral common carotid artery occlusion
BSCB: blood-spinal cord barrier
CBF: cerebral blood flow
CCH: chronic cerebral hypoperfusion
CNS: central nervous system
CNX: calnexin
DAPI: 4 ′,6-diamidino-2-phenylndole
DG: dentate gyrus
DI: discrimination index
DMSO: dimethyl sulfoxide
ER: endoplasmic reticulum
ERMES: endoplasmic reticulum mitochondria encounter structure
ERp44: ER resident protein 44
FTD: frontotemporal dementia
GRP75: glucose-regulated protein
HD: Huntington’s disease
IP3Rs: inositol-1,4,5-triphosphate receptors
LTD: long-term depression
LTP: long-term potentiation
MAMs: mitochondria-associated endoplasmic reticulum membranes
MCSs: membrane contact sites
MFN1: mitofusion1
MFN2: mitofusion2
MWM: Morris water maze
NVU: neurovascular unit
OFT: open field test
ORT: object recognition test
PACS-2: phosphofurin acidic cluster sorting protein-2
PD: Parkinson’s disease
PDI: protein disulfide isomerase
PSCI: poststroke cognitive impairment
PSD: poststroke depression
PTPIP51: mitochondrial protein tyrosine phosphatase-interacting protein-51
PVC: polyvinyl chloride
SD rats: Sprague-Dawley rats
TEM: transmission electron microscopy
TOMM20: outer mitochondrial membrane protein-20
TRPV1: transient receptor potential vanilloid type 1
VAPB: VAMP-associated protein B
VCI: vascular cognitive impairment
VDAC1: voltage-dependent anion channel
VR1: vanilloid receptor subtype 1
WMLs: white matter lesions.
