# Supplementary material for: Capsaicin Ameliorates the Loosening of Mitochondria-Associated Endoplasmic Reticulum Membranes and Improves Cognitive Function in Rats With Chronic Cerebral Hypoperfusion
Source: Front Cell Neurosci. 2022 Mar 17;16:822702. doi: 10.3389/fncel.2022.822702 (PMC8968035; doi:10.3389/fncel.2022.822702)
Supplement: Supplementary file 1 [file Data_Sheet_1.docx]

Supplementary Material

# Supplementary Figures and Tables

## Supplementary Figures


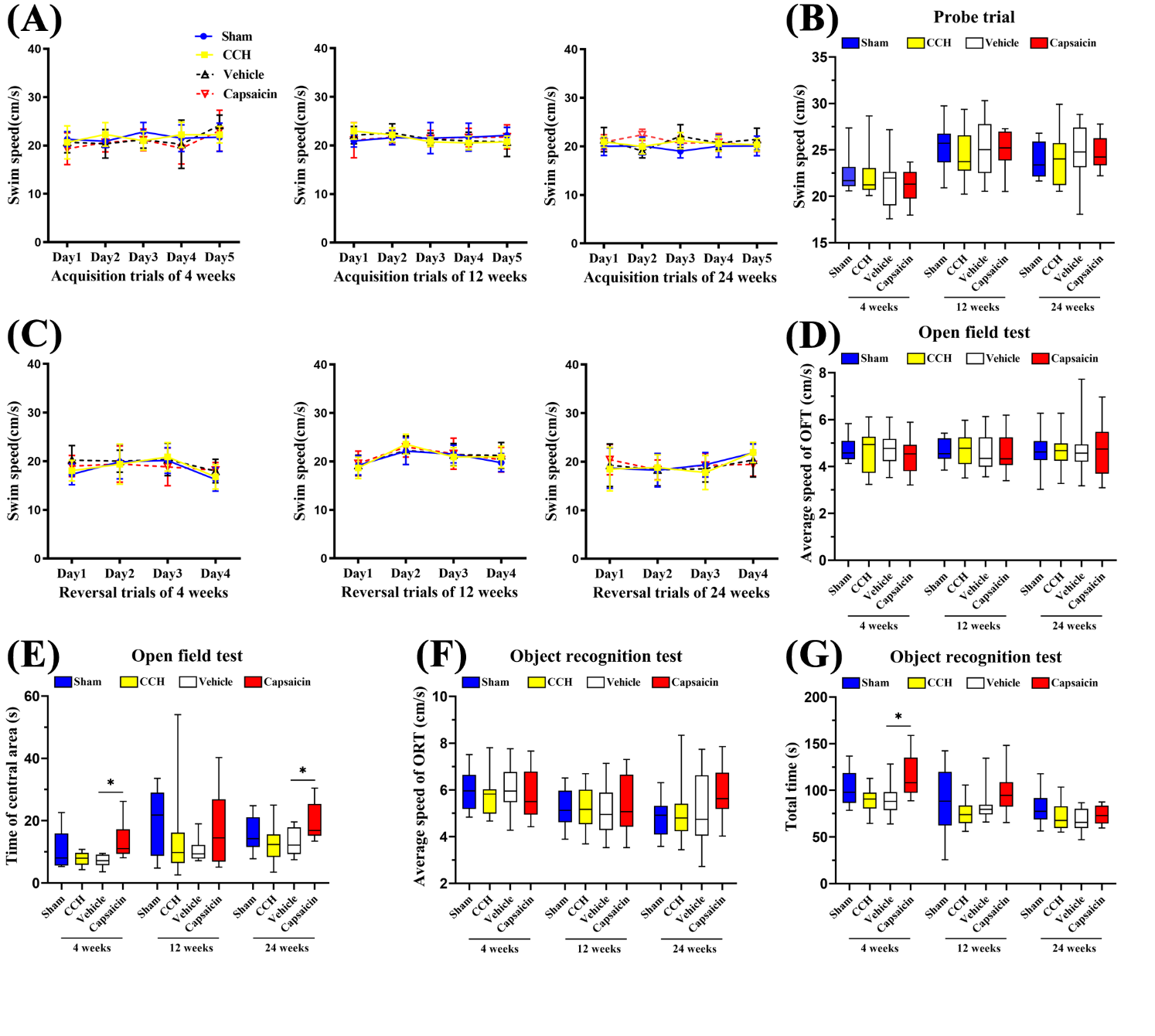


**Supplementary Figure 1.** **(A)** Swimming speed of each group in acquisition trials of MWM. **(B)** Average speed of each group in probe trials of MWM. **(C)** Swimming speed of each group in reversal trials of MWM. **(D)** Average speed of each group in OFT. **(E)** The time spent traveled in the central area of OFT. **(F)** Average speed during the ORT. **(G)** Total exploration time in the recognition phase of ORT. Data are presented as the mean ± SD. **P* < 0.05. Data of swimming speed in acquisition trials and reversal trails of MWM were analyzed by two-way ANOVA followed by the *post hoc* LSD test. Data of swimming speed in probe trials of MWM were analyzed by unpaired t-test. Data of ORT and OFT were analyzed by one-way ANOVA followed by the *post hoc* LSD test.

## Supplementary Tables

| Resource Type | Specific Reagent or Resource | Source and Identifiers | Additional Information |
| --- | --- | --- | --- |
| Antibody | mouse anti-mitofusin2 | Abcam, Cat. No. ab56889, RRID: AB_2142629 | dilution factor (WB): 1:500 |
| Antibody | mouse anti-GAPDH | Abcam, Cat. No. ab8245, RRID: AB_2107448 | dilution factor (WB): 1:1000 |
| Antibody | horseradish peroxidase-conjugated goat anti-mouse IgG | Proteintech, Cat. No. SA00001-1, RRID: AB_2722565 | dilution factor (WB): 1:2000 |
| Antibody | rabbit anti-translocase of the outer mitochondrial membrane protein-20 (TOM20) | Abcam, Cat. No. ab78547, RRID: AB_2043078 | dilution factor (I.F.): 1:200 |
| Antibody | mouse anti-protein disulphide isomerase (PDI) | Abcam, Cat. No. ab2792, RRID: AB_303304 | dilution factor (I.F.): 1:250 |
| Antibody | AlexaFlour 488/594 | Invitrogen | dilution factor (I.F.): 1:1000 |
| Drug | pentobarbital sodium | Sigma, Cat. No. P3761 |  |
| Drug | capsaicin | Alomone Labs, Cat. No. C-125 | vehicle solution: sterile 0.9% saline, DMSO, and Tween 80 (80/10/10%, V/V) |
| Chemical Compound | sterile 0.9% saline | Beyotime, Cat. No. ST038 |  |
| Chemical Compound | dimethyl sulfoxide (DMSO) | Beyotime, Cat. No. ST341 |  |
| Chemical Compound | Tween-80/Tween-20 | Solarbio, Cat. No. T8360/T8220 |  |
| Chemical Compound | paraformaldehyde | Boster, Cat. No. AR1069 |  |
| Commercial Assay Or Kit | protein extraction kit | Solarbio, Beijing, Cat. No. BC3711 | WB |
| Commercial Assay Or Kit | BCA protein assay kit | Beyotime, Shanghai, Cat. No. P0012S | WB |
| Chemical Compound | opti-mum cutting temperature compound | SAKURA, USA | I.F. |
| Chemical Compound | osmium tetroxide | Beijing Zhongjingkeyi Technology Co., Ltd. Cat. No. GP18456 |  |
| Chemical Compound | epoxy resin (Epon 812) | Beijing Zhongjingkeyi Technology Co., Ltd. Cat. No. GP18010 |  |
| Chemical Compound | uranyl acetate | Beijing Zhongjingkeyi Technology Co., Ltd. Cat. No. GS02624 |  |
| Chemical Compound | lead citrate | Beijing Zhongjingkeyi Technology Co., Ltd. Cat. No. GZ02616 |  |
| Chemical Compound | glutaraldehyde | Solarbio, Cat. No. P1127 |  |
| Chemical Compound | TBS/TBST | Solarbio, Cat. No. T1080/T1081 |  |
| Chemical Compound | PBS | Solarbio, Cat. No. P1010 |  |
| Equipment | Light microscope | Olympus, IX81 | numerical aperture (NA): 40x 0.6, 20x 0.45,10x 0.3, 4x 0.13 |
| Equipment | Confocal microscope | Nikon, A1R+ | numerical aperture (NA): 60x 0.75, 40x 0.65, 20x 0.40,10x 0.25, 4x 0.10 |

**Supplementary Table 2**. Key resources table.
